# European projections of West Nile virus transmission under climate change scenarios

**DOI:** 10.1016/j.onehlt.2023.100509

**Published:** 2023-02-16

**Authors:** Zia Farooq, Henrik Sjödin, Jan C. Semenza, Yesim Tozan, Maquines Odhiambo Sewe, Jonas Wallin, Joacim Rocklöv

**Affiliations:** a Department of Public Health and Clinical Medicine, Section of Sustainable Health, Umeå University, Sweden; b Heidelberg institute of global health and Interdisciplinary center for scientific computing, University of Heidelberg, Im Neuenheimer Feld 205, Heidelberg 69120, Germany; c School of Global Public Health, New York University, New York, USA; d Department of statistics, Lund university, Sweden

**Keywords:** West Nile virus, Zoonoses, Europe, XGBoost, WNV risk projections, Climate change, Climate impacts, Artificial intelligence, Confidence-based performance estimation (CBPE) method

## Abstract

West Nile virus (WNV), a mosquito-borne zoonosis, has emerged as a disease of public health concern in Europe. Recent outbreaks have been attributed to suitable climatic conditions for its vectors favoring transmission. However, to date, projections of the risk for WNV expansion under climate change scenarios is lacking. Here, we estimate the WNV-outbreaks risk for a set of climate change and socioeconomic scenarios. We delineate the potential risk-areas and estimate the growth in the population at risk (PAR).

We used supervised machine learning classifier, XGBoost, to estimate the WNV-outbreak risk using an ensemble climate model and multi-scenario approach. The model was trained by collating climatic, socioeconomic, and reported WNV-infections data (2010−22) and the out-of-sample results (1950–2009, 2023–99) were validated using a novel Confidence-Based Performance Estimation (CBPE) method. Projections of area specific outbreak risk trends, and corresponding population at risk were estimated and compared across scenarios.

Our results show up to 5-fold increase in West Nile virus (WNV) risk for 2040-60 in Europe, depending on geographical region and climate scenario, compared to 2000-20. The proportion of disease-reported European land areas could increase from 15% to 23-30%, putting 161 to 244 million people at risk.  Across scenarios, Western Europe appears to be facing the largest increase in the outbreak risk of WNV. The increase in the risk is not linear but undergoes periods of sharp changes governed by climatic thresholds associated with ideal conditions for WNV vectors. The increased risk will require a targeted public health response to manage the expansion of WNV with climate change in Europe.

## 1 Introduction

West Nile virus (WNV) is a climate sensitive, multi-vector, multi-host zoonosis, with mosquitoes serving as vectors and birds as the principal, amplifying hosts [1]. To date, WNV circulates on many continents, including Africa, Americas, Australia and Europe, where predominantly Southern and Central countries are affected, such as Romania and Italy [2,3], human infections of WNV were mainly associated with sporadic cases up to the mid-1990s [4]. During the last few decades, Europe has experienced an upsurge in the recurrence, a geographic expansion of the outbreak areas, and a higher incidence associated with WNV-outbreaks, particularly in 2010 and 2018 [[5], [6], [7], [8]].

The epidemiology and transmission of WNV is very intricate and depends on both abiotic and biotic factors [9]. Among the abiotic factors, temperature appears to be the most critical driver of transmissions, but precipitation, land-use and physical environmental features are also important [9,10]. Significant biotic factors of WNV transmission include host abundance and diversity, vector distribution and bird migration.

Empirical studies have established ambient temperature to be one of the most significant determinants of WNV activity in Europe [[9], [10], [11], [12]]. Ambient temperature determines mosquito survival, vector population growth rates, the intervals between blood meals, as well as the extrinsic incubation period (EIP) through its effect on viral replication rates [[13], [14], [15], [16]]. It can also accelerate viral replication rates in vectors and speed up the frequency of vector–host interaction ultimately increasing biting rates [16,17].

Among a wide array of eco-climatic drivers, climatic anomalies, for instance summer and spring temperatures during last decade, were overwhelmingly the strongest determinants of recurrent WNV-outbreaks in Europe [11]. Advanced data-driven machine learning algorithms quite accurately predicted the regions (NUTS3: Nomenclature unit for terrestrial statistics, at level 3) of WNV-outbreaks with eco-climatic predictors. NUTS is a geographical division of European union at three hierarchical levels where NUTS3 correspond to the smallest (i.e., to district-level) units [18]. Notably, the unusual spring temperatures during the year 2018 were one of the main driver of extraordinary WNV-outbreak that year [11].

These findings are important considering changing climate that is projected to further increase the frequency, intensity, and duration of extreme weather events, such as heatwaves, floods, or droughts. Such events can not only amplify the interaction between vector and host but also vectors and viruses which favor the WNV transmission to humans, resulting in more outbreaks [19,20]. Specifically, ambient temperature is expected to increase between 1.0 °C and 5.5 °C by the end of the century [21]. Increases in temperature due to climate change are therefore projected to impact the WNV transmission also in currently risk-free European parts [22]. Elucidating the spatiotemporal expansion of WNV across Europe under different climate change scenarios has tangible public health implications, such as protecting blood supplies in affected areas [23]. Therefore, projecting the risk of WNV-outbreaks using these climatic determinants can help direct preparedness efforts and public health measures to targeted efforts as outbreak risk areas change.

Data-driven models with high predictive ability are considered important tools for this purpose. These models estimate the disease risk by searching the association patterns in collated spatiotemporal predictors and response (disease records) data. With their proven applicability to diverse fields across science [[24], [25], [26], [27]], and with ever-increasing computational power and data availability, these algorithms have proven to be a useful tool to conduct such analyses. The advantage of these algorithms is that they require no specific assumptions on parameters representing, e.g., climatic, and biological processes, in contrast to process-based models. They also lend themselves well to robustly capture higher order interactions and non-linear relationships.

Advancement in climate science additionally now make it possible to project climate-change-induced WNV-outbreak risk based on a rich set of bias corrected ensemble climate models, consisting of carbon (CO_2_) concentration forcing, coupled with future scenarios and represent a set of possible socioeconomic paths.

In this study, therefore, we used a machine learning classifier, XGBoost, to estimate and project the WNV-outbreaks risk for Europe. We did this by collating climatic, and socioeconomic predictors data with an ensemble climate and multi-scenario approach. We first trained our model for each climate data set separately against reported WNV-infections data (2010−22) at the district (NUTS3) level for Europe. We then projected the WNV-outbreak risk (2023–99) and validated our results using a novel binary classification method, CBPE. We estimated the historic risk for the period 1950–2009 to quantify the change in the relative risk. We then delineated and mapped potential WNV risk-areas and quantified their population at risk (PAR) under each scenario stratified by European regions.

## 2 Methods

### 2.1 Data

Spatiotemporal climate data representing three combined shared socioeconomic pathways (SSP1,3, and 5) and representative concentration pathways (RCP; 26, 70, and 85) were retrieved from bias-corrected ISIMIP3b (Inter-Sectoral Impact Model Intercomparison Project, phase-3) based on output of CMIP6 (Coupled Model Intercomparison Project, phase 6) [[28], [29], [30]]. These data represent possible climate futures based on, combined effects of greenhouse gas and other anthropogenic factors that might influence climate and are labelled based on their end-of-century radiative forcing relative to preindustrial conditions (e.g., RCP26 represents a 2.6 W/m^2^ increase).

Here, to account for uncertainties, we considered a range of scenarios ranging from very stringent (RCP26) to business-as-usual (RCP 85) in terms of emissions and radiative forcing. Specifically, SSP1-RCP26 represents low challenges to mitigation and adaptation in SSP1 with low CO_2_ emissions. SSP3-RCP70 represents medium-high reference scenario within the “regional rivalry” socio-economic, and high CO_2_ emissions. Lastly, SSP5-RCP85 represents extremely high challenges to mitigation with low challenges to adaption, and extremely high CO_2_ emissions.

The climate data of daily surface temperature (in K), total precipitation (in kg/m^2^ per second) and relative-humidity (%) were aggregated spatially at NUTS3 level and were divided into four quarters per year [29]. Further, to enhance the model's predictive power a set of 19 bioclimatic predictors (bio01-bio19) [31], that delineate the annual trends were also incorporated.

For historical runs, we used E-OBS climate data set from 1950 to 2014 [32]. The data from 2015 to 2099 were obtained from five primary general circulation models (GCMs) of CMIP6 in order to account for uncertainties in estimates (see Table 1).

**Table 1:** Summary of climate scenarios, climate models, time-period, and predictors/response.

**Climate Scenario**	**Climate Model**	**Period**	**Predictors/ response**
Historical	E-OBS	1950‐2014	i) Temperature (Q) (min, mean, max) ii) Total precipitation (Q) iii) Total relative humidity (Q) iv) Bioclimatic (bio1: bio19) v) Population density (A) vi) Gross domestic product (A) vii) NUTS3 WNV infections (A)
SSP1-RCP26	i) IPSL-CM6A-LR ii) MPI-ESM1‐2-HR iii) MRI-ESM2‐0 iv) UKESM1‐0-LL v) GFDL-ESM4	2015‐2099	
SSP1-RCP70			
SSP3-RCP85			

Q = quarterly predictors, A = annual predictors.

Population densities, often used as a proxy for urbanization, and socioeconomic factors, are also associated with increased risk of WNV transmission [11]. We, therefore, also incorporated socioeconomic predictors, in order to account for their impact on disease risk predictions. Specifically, scenario-wise data for annual population density and gross domestic product (GDP) were also added predictors [33].

For the response variable, the data on West Nile virus infection notifications at NUTS3 level (2021 definition) were obtained from the *European Center for Disease Prevention and Control* (ECDC) (see Appendix; WNV infections data) [34]. The influence of the predictors was presented by dividing regions into two classes based on annual WNV infection activity [11]. The regions reporting at least one reported infection were labelled as WNV positive (1), and negative (0) otherwise.

### 2.2 Machine learning algorithm

We used a supervised machine learning classifier, XGBoost, for all the analyses [35]. As described previously on the methodology [11], the algorithm showed a high predictive ability to classify a region with WNV outbreak using eco-climatic predictors as the most prominent features for the discrimination of classes. Briefly, here we used a *10*-fold cross-validation strategy for the model training and hyperparameters tuning. The model fit, internal validation, and tuning process were computed using the *logloss-score*. The model hyperparameters were optimized using a random search approach to infer the best fit [11]. The model with the lowest *logloss-score* was chosen for analysis. The details on cross-validation and hyperparameter tuning can be found in the appendix (see ‘Model cross-validation and hyperparameter tuning’).

### 2.3 Out-of-sample performance evaluation

**Fig. 1:**
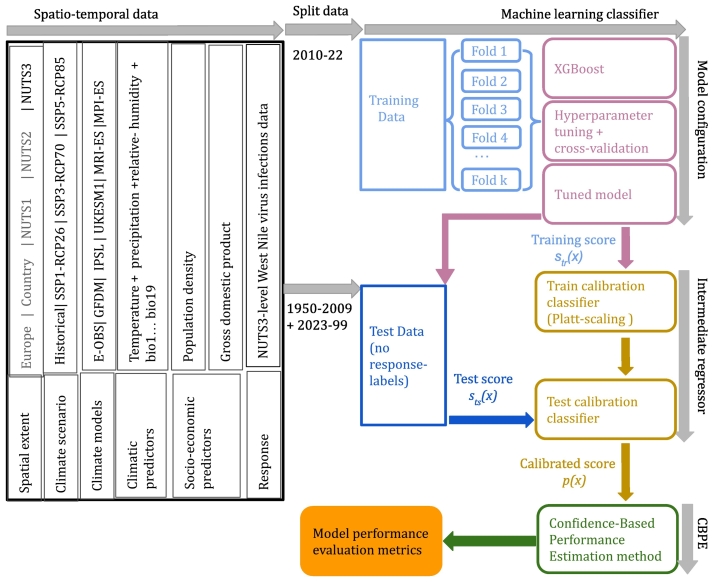
Spatio-temporal data collation and modelling setup. The spatio-temporal data consisting of climatic and socio-economic predictors for a climate-scenario from each climate-model was collated with the West Nile virus infections data at NUTS3 level. Every instance of a data set was divided into training (2010–22) and test (1950–2009, 2023–99) periods, respectively. Then, a machine learning classifier, XGBoost, was trained with a 10-fold cross-validation and hyperparameter tuning. Then the training scores (*s*_*tr*_(*x*)) were passed to an intermediate calibration classifier (here Platt-scaling) [40]. Once tuned, these uncalibrated scores (*s*_*ts*_(*x*)) of test data were estimated with tuned XGBoost. These scores were provided as input to the trained calibration classifier that produced calibrated scores (*p*(*x*)) of the test data. Finally, Confidence-Based Performance Estimation (CBPE) method was applied on calibrated test scores to evaluate the model performance metrics on test data without ground-truth.

Generally, a machine learning classifier's performance on out-of-sample data can be measured either with threshold-independent metrics (such as receiver operator characteristic (ROC) curve) or with threshold-dependent confusion-matrix related scores provided the ‘ground-truth’ (i.e., actual class-labels) is available. But what if the ground-truth is absent which is the case here? Should one rely on model's performance metrics of internal validation period to make projections? One caveat of these classifiers is that their performance remains consistent if the distributions of the training and test data of their predictors are statistically similar (i.e., there is no data-drift) and the association between the predictors and response remains similar (i.e., no concept-drift) [36,37]. However, if there is a data-drift, one may face the challenge of deteriorated but latently unaccounted performance of a classifier. This may hold true when considering the distributions of historic, current, and future climate data. Thus, it then becomes critical to ensure that a classifier's performance in the absence of ground-truth on out-of-sample data is reliable. Fortunately, it is possible now with a novel approach; Confidence-Based Performance Estimation (CBPE) method implemented in open-source Python library, NannyML [37,38]. The method can provide expected performance of a classifier even in the absence of ground-truth in the presence of data-drift. The expected performance of classifier is measured by just providing its calibrated scores as input to the CBPE method assuming that there is no concept-drift (Fig. 1) (see Appendix for details).

To this end, we examined the XGBoost classifier's performance on out-of-sample data with various metrics estimated using the CBPE method. These metrics, include AUROC-score (area under ROC-curve), AUPRC-score (area under precision-recall characteristic (PRC)-curve) and threshold-dependent metrics (*sensitivity*, *specificity* etc.,).

The threshold-dependent metrics were estimated at optimal classification-thresholds from ROC (and PRC)-curves. The optimal classification-threshold from ROC-curve was estimated using the index of union (IU) method that considers the maximum sensitivity and specificity scores [39]. Similarly from PRC-curve it was estimated using *F1-score* which is the harmonic mean of *precision* and *recall* metrics.

The threshold from PRC-curve was used to classify if a region was at risk or not. This was given preference over commonly used ROC-curve in order to control for the model's false discovery rate. The model classified risk areas provided the basis to quantify the population at risk (PAR). Simplistically, the population at risk was defined as the population size of an area that was classified as WNV positive by the model. These estimates were computed for the two future periods of 2040–60 and 2080–00 and were compared with the baseline period of 2010–22.

## 3 Results

### 3.1 West Nile virus outbreak risks

**Fig. 2:**
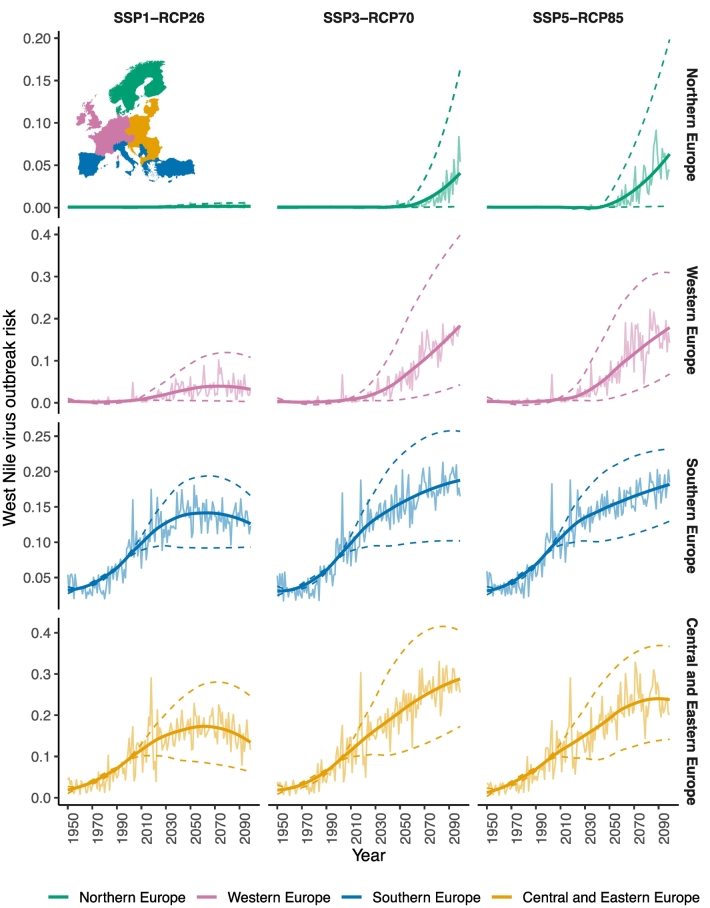
West Nile virus risk trends under climate change scenarios. Year-to-year West Nile outbreak risk trends for European regions under each scenario from 1950 to 2100 predicted by the machine learning algorithm, XGBoost. SSP1-RCP26 represents low challenges to mitigation and adaptation and low CO_2_ emissions, SSP3-RCP70 represents medium-high reference scenario within the “regional rivalry” socio-economic and high CO_2_ emissions, and SSP5-RCP85 represents extremely high challenges to mitigation with low challenges to adaption and extremely high CO_2_ emissions. The horizontal axis represents the year of predictions. The vertical axis shows the corresponding model predicted risk of West Nile virus. Mean annual estimates and variations in the risk estimates from all the climate models are shown by the smooth regression dashed-curves. The map in the inset figure shows the legend for these regions.

The WNV risk is projected to expand beyond the currently infected areas in the coming decades across all scenarios, most notably to Western Europe (Fig. 2, Western Europe). For Europe, the risk could increase up to 3.5-fold, even under the most conservative scenarios during 2040–60, compared to 2000–20. The risk may increase by 2.5-fold (SSP1-RCP26) to 5-fold (SSP5-RCP85) during 2040–60 for Western Europe. While no substantial change in risk is expected under SSP1-RCP26 for Northern Europe (Fig. 2), it is projected to increase considerably (4.8-fold) under SSP5-RCP85. For Central and Eastern Europe, which are currently endemic, the risk could increase by 0.3-fold under SSP1-RCP26 and 0.7-fold under extreme scenarios during the same period. For Southern Europe, the projected increase in risk is estimated to be between 0.4-fold to 0.5-fold under low to extreme scenarios.

### 3.2 Potential areas at West Nile virus risk

**Fig. 3:**
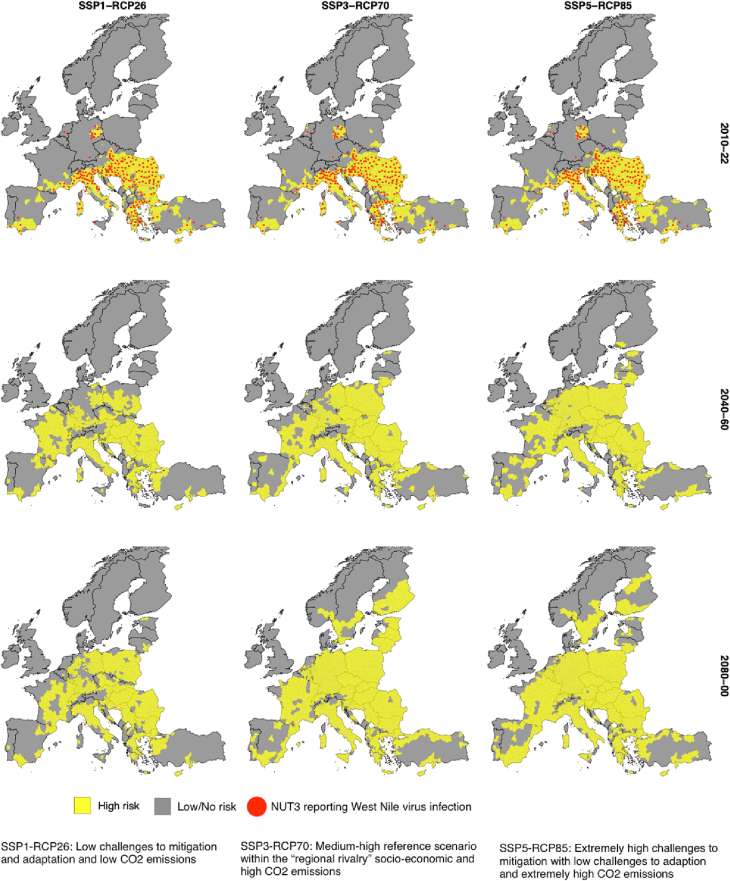
West Nile virus risk maps under climate-change scenarios. From top to bottom, the maps show the reported and the model predicted WNV risk areas under each climate scenario of the baseline period (first-row) and two future periods (2nd and 3rd row, respectively). The model classified ‘High risk’ (yellow) regions and ‘Low/No risk’ (grey) regions are aggregated from all the climate models for the given periods. The overlaid red-dots indicate regions reporting West Nile virus infections during the baseline period. Countries' boundaries are distinguished by black color.

**Fig. 4:**
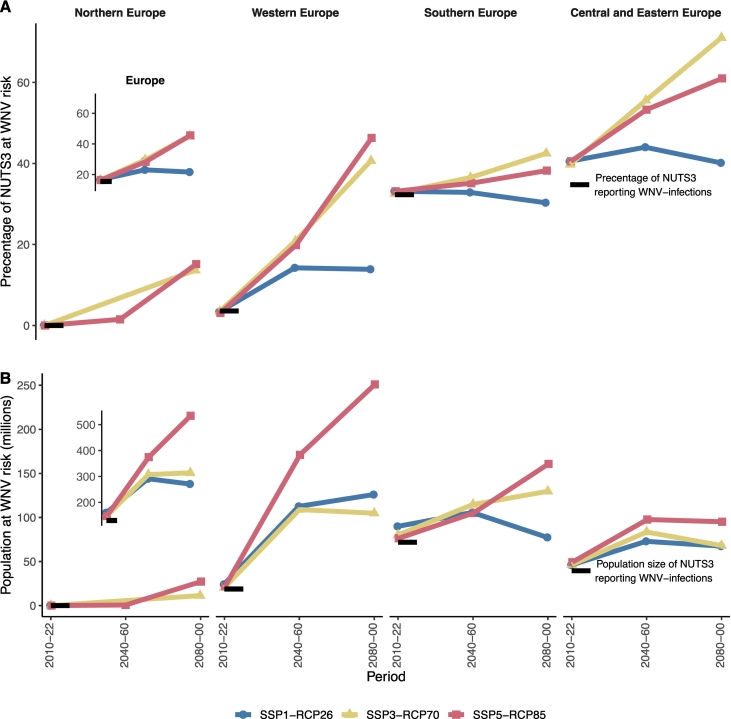
**A)** New regions at West Nile risk Comparison of model projected regions (%) at West Nile virus risk under each climate scenario stratified by European regions corresponding to the baseline period (2010-22). SSP1-RCP26 represents low challenges to mitigation and adaptation and low CO_2_ emissions, SSP3-RCP70 represents medium-high reference scenario within the “regional rivalry” socio-economic and high CO_2_ emissions, and SSP5-RCP85 represents extremely high challenges to mitigation with low challenges to adaption and extremely high CO_2_ emissions. The bold black line shows the percentage of regions reporting the West Nile virus infections during the baseline period and colors stand for the model predictions for each climatic scenario. The inset figure represents the estimates for whole Europe. **B)** Population at risk estimates: Comparison of population at risk estimates under each climate scenario stratified by European regions corresponding to the baseline period (2010-22). The bold black line represents population size of regions reporting the WNV-infections during the baseline period. The inset figure indicates the PAR estimates for whole Europe.

We mapped and compared the areas reporting WNV infections with the model predicted risk-areas under each scenario during the baseline period (Fig. 3, 2010–22). We also show the model predicted risk areas for two future periods representing spatiotemporal expansion of the virus (Fig. 3). The potential risk-areas, on average, increase to 23% (SSP1-RCP26) to 29% (SSP5-RCP85) during 2040–60 (Fig. 4A, inset), when compared with baseline proportion (15.4%) of disease-reported European land areas. These estimates may further proliferate to 46% late this century (SSP5-RCP85) where the outbreak risk-areas are predicted to expand to Northern Europe (Fig. 2, 2080–00).

In Fig. 4A, the estimates of model projected risk-areas are shown and compared with the baseline period. The increase in risk for Western Europe is the most striking one (up to 10-fold) in terms of geographical-expansion. During 2040–60, the risk-areas may increase to 14% (SSP1-RCP26) to 31% (SSP7-RCP70), when compared with baseline proportion of 3.6%. For Central and Eastern Europe, the current proportion (35%) of areas reporting WNV transmission, is projected to increase to 44% (SSP1-RCP26) to 56% (SSP3-RCP70). Similarly, for Southern Europe, the current proportion (32%) of infected areas could increase to 33% (SSP1-RCP26) to 39% (SSP3-RCP70) during this period. Potential risk-areas in currently risk-free Northern Europe are approximated to be 2% (SSP5-RCP85) during the same period (Fig. 4A).

### 3.3 Populations at West Nile virus risk

A comparison of projected estimates of PAR across scenarios for the defined periods are given in Fig. 4B. Overall, for Europe, we estimated an additional 161 to 244 million people at risk during 2040–60 depending on climate scenarios (Fig. 4B, inset). More densely populated Western Europe is projected to have the largest proportion of increased PAR irrespective of any climate scenario. Approximately 94 million people could be at risk during 2040–60 even under most conservative scenario (SSP1-RCP26) in this region (Fig. 4B). Similarly, the additional PAR is projected to be 33 million for Central and Eastern Europe, 34 million for Southern Europe and 0.7 million for the same period. For the same period 0.7 million people in Northern Europe are projected to be at risk but only under SSP1-RCP85.

### 3.4 Spring climate and West Nile virus outbreaks

**Fig. 5:**
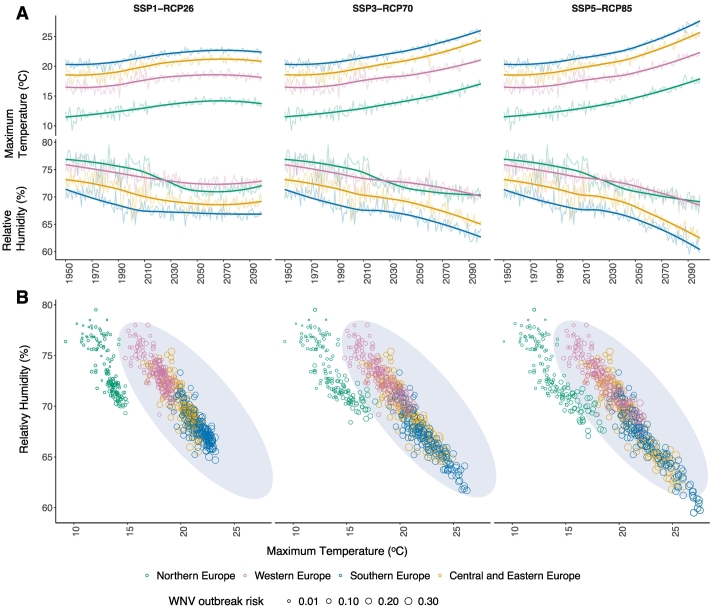
WNV-outbreak risk as function of spring temperature and relative humidity. SSP1-RCP26 represents low challenges to mitigation and adaptation and low CO_2_ emissions, SSP3-RCP70 represents medium-high reference scenario within the “regional rivalry” socio-economic and high CO_2_ emissions, and SSP5-RCP85 represents extremely high challenges to mitigation with low challenges to adaption and extremely high CO_2_ emissions. **A)** Maximum spring temperature and the relative humidity under each climate scenario stratified by European region wise. **B)** The model predicted West Nile virus outbreak risk as function spring climatic conditions. The horizontal axis represents the maximum spring temperature, the vertical axis represents relative humidity of the spring season. The bubble sizes correspond to the amount of predicted risk, the larger the bubble size, the higher the risk. The ellipses represent the parameteric space with increased risk.

Our analysis revealed that increased risk is not linear but undergoes periods of sharp changes governed by climatic thresholds associated with ideal conditions for its vectors. We found a strong positive association between maximum spring temperature (ρ∼0.8) and the risk, and strong negative association (ρ∼‐0.7) between relative humidity and outbreak risk (Fig. 5). Specifically, the risk increased substantially when spring temperature was >15°C (lower threshold) and relative humidity was <77% (upper threshold), respectively across all regions and scenarios (Fig. 5B). The pattern can be clearly observed for Northern Europe when comparing SSP1-RCP26 to SSP5-RCP85 (Fig. 5B). These climatic threshold have been associated with ideal conditions for disease vectors [41].

## 4 Discussion

This is the first European study collating climatic and socioeconomic scenarios to estimate WNV risk projections using a bias-corrected ensemble climate-model and multi-scenario approach. A machine learning classifier, XGBoost, was applied to a suite of general circulation models representing three likely socioeconomic, and CO_2_ emissions pathways to quantify the WNV expansion risk. The projected estimates were validated using another a novel classification algorithm, CBPE, with various metrics (Appendix; Performance metrics on out-of-sample data). All the projections showed a higher WNV risk associated with elevated spring/summer temperatures and drier conditions. Overall trends showed a North-Western dispersal of the virus in the 2nd half of this century under extreme scenarios. Western Europe, in particular, seems to be facing larger risk of WNV-outbreaks in the near and distant future.

### 4.1 Spring climate-early warning signal

West Nile vectors usually show optimal activity between the spring and autumn seasons in Europe while the majority of WNV infections in humans and equids have been reported in the 3rd quarter of the year [7]. The spring temperature as shown previously [42,43], and very recently [9], seems to shape the virus transmission in Europe. Our findings highlight that the spring climatic conditions remain an important precursor of the potential WNV-outbreaks. This is evidenced by a strong positive association (ρ∼0.8) between maximum spring temperatures and an increased outbreak risk with a threshold of >15 °C. Similarly, relative humidity of the spring was found to have a strong negative association with disease risk (ρ ∼‐0.7) (Fig. 5B). As increase in temperature leads to a decrease in relative humidity; thus, a negative association between the outbreak risk and the relative humidity seems reasonable.

Using climate signals as WNV transmission alert could be an efficient source compared to using entomological surveillance data of the mosquitoes or the host birds that require a considerable amount of time, effort, and resources. Thus, when developing climate-related WNV early warning systems, the spring climate parameter should be given priority.

### 4.2 Public health concerns of West Nile virus expansion

Increased WNV distribution and disease burden have both direct and indirect implications for Europe from a public health perspective. For instance, among others, the virus is transmitted through blood-transfusion. Due to its high asymptomatic proportion of disease and the potential of transmission through blood-transfusion, higher prevalence in blood-donor population has been estimated for WNV-affected European regions [22]. Hence, dispersal to new areas will be an additional concern to the safety of blood banks. Another public health concern is that these populations at risk will now also be at risk for West Nile neuroinvasive disease (WNND) which will require intensive care, to avoid fatalities.

Further, evidence of a strong association between agricultural activities and WNV incidence [44], an economic downturn is also reported in the literature [12]. Assessing the implications of economic crises, agricultural activities, urbanization and health economics metrics, such as disability adjusted life years, can provide valuable insights to quantify the actual disease burden in Europe. We omit these analyses here for future work.

Early detection of a disease outbreak can accelerate the public health response and reduce the risk to individuals, the economy, and society at large. Insights from the presented analyses could be of vital interest for policy decision support tools, such as the European Climate and Health Observatory [45], created as part of the European Green Deal [46], the EU Adaptation Strategy [47], and EU4Health [48]. These results can guide targeted surveillance efforts to more proactive identification of WNV in vector or host animals [49]. Currently, areas adjacent to currently infected NUTS3 regions, are at higher risk as predicted by our model (Fig. 2, 2010–22). Therefore, these areas should be considered a priority for public health interventions and vector control [49].

### 4.3 Limitations

While the presented analysis includes both climatic and non-climatic determinants of WNV transmission, other drivers, such as interventions to mosquitoes and future land use changes were not included. The contribution of these measures is difficult to evaluate and quantify, as projections are lacking for future scenarios. Also, as noted above, there are various complex intrinsic constituents of the WNV transmission cycle including amplifying host birds and mosquitoes vectors which appear to differ between continents and setting. Further on, the response of biological, vectors and birds ecology to climate could further differ from what is assumed in these projection [9]. For example, information on routes of migratory birds and how they are affected by climate change would be of interest. Similarly, data on equine and avian WNV cases are also important as their reporting differ from human cases. Thus, future work may focus on including a multitude of host data for improved risk assessments. This, in essence, requires more transdisciplinary research to make projections of other important drivers of WNV transmission, in addition to those considered here [50].

## 5 Conclusion

WNV, already endemic in parts of European countries, will likely continue to disperse to naive areas, as conditions for its vectors become more favorable due to the changing climate. Western Europe could be facing large outbreaks of the virus, irrespective of the future degree of climate change, calling for the need to adapt to this new situation. Under high emission scenarios, WNV could even expand to Northern Europe later in this century.

Concrete and well-coordinated efforts are needed given the increasing significance of WNV for European public health. Early warning systems, vector and disease surveillance, vector control measures, health promotion and awareness raising among the public and health care providers are crucial components of an effective public health response [49]. All these actions call for inter-sectorial collaboration, cross-disciplinary research, and using advanced disease modelling to inform targeted actions.

## Author statement

Conceptualization: JR, JCS, ZF and JW conceptualized the idea of the study.

Data collection and data curation: ZF, MSO, YT and HS curated the data. ZF synthesized all the data from various sources and set up software codes.

Formal Analysis, investigation, and methodology: ZF conducted the formal analysis, implemented the investigation and methodology. JW, JR, HS and JCS provided expert support for the analysis.

Writing-original draft: ZF wrote the initial draft. JCS, JR, YT and HS provided technical guidance and contributed to writing the final draft.

Reviewing & editing: All the authors were involved in critically reviewing, editing, and agreed to the final version of the manuscript.

### Data credit

Data on West Nile virus infections from The European Surveillance System – TESSy, provided by Austria, Bulgaria, Cyprus, Czech Republic, Germany, Greece, Spain, France, Croatia, Hungary, Italy, Netherlands, Portugal, Romania, Serbia, Slovenia, Slovakia and Turkey and released by ECDC.

### Disclaimer

The views and opinions of the authors expressed herein do not necessarily state or reflect those of ECDC. The accuracy of the authors’ statistical analysis and the findings they report are not the responsibility of ECDC. ECDC is not responsible for conclusions or opinions drawn from the data provided. ECDC is not responsible for the correctness of the data and for data management, data merging and data collation after provision of the data. ECDC shall not be held liable for improper or incorrect use of the data.

## Declaration of Competing Interest

All authors declare no competing interests.

## Data Availability

The WNV case data can be requested from ECDC. All other data are open access.

## Appendix A Supplementary data

Supplementary material Image 1
