# Supplementary material for: European projections of West Nile virus transmission under climate change scenarios
Source: One Health. 2023 Feb 16;16:100509. doi: 10.1016/j.onehlt.2023.100509 (PMC10288058; doi:10.1016/j.onehlt.2023.100509)
Supplement: Supplementary file 1 — Supplementary material [file mmc1.docx]

Appendix

European projections of West Nile virus transmission under climate change scenarios

Zia Farooq^a^, Henrik Sjödin^a^, Jan C. Semenza*^a,b^, Yesim Tozan^c^,

Maquines Odhiambo Sewe^a^, Jonas Wallin^d^, Joacim Rocklöv^a,b^

# ****West Nile virus infections data****

Human WNV notifications for the years 2006 to 2022 were obtained and extracted from the *European Center for Disease Prevention and Control* (ECDC) and were aggregated by NUTS3 regions.^1^ Both asymptomatic and human cases with neuroinvasive WNV disease were included in the analysis. Besides the latter being more severe presenting with meningitis, encephalitis, and acute flaccid paralysis, the former constitutes the majority (about 80%) of the WNV infections.^2^ Infections with unknown aetiology and the imported infections were excluded from the analyses. We restricted the training of the model for the years 2010-22. The proportion of positive instances (i.e., regions with WNV transmission) in the data set for the period 2006-09 was just ~ 0.2% inclusion of which further skewed the class-imbalance of the internal validation period. The selected training data contained a total of 810 NUTS3 level observations of recorded WNV transmission included in the main analysis.

The WNV amplifying host birds and animals infections data at NUTS3 level was available with spatial information only and not with any temporal identity except for the year 2022, that was extracted from the ECDC dashboard source (<https://gis.ecdc.europa.eu/portal/apps/experiencebuilder/experience/?id=4876503d343a4c1abf5941557eb071f1> ). Hence, we didn’t make them part of the analyses, though their inclusion can further strengthen the analyses.

# Spatiotemporal data extraction and aggregation

# All the spatiotemporal climatic and non-climatic data was aggregated at NUTS3 level. The NUTS3 is the highest spatial classification system divided based on European economic regions after NUTS1 and NUTS2. All spatial information was captured at the NUTS3 level using shapefiles (polygons) sourced from ‘*eurostat’* package of R software.^3,4^ We used the 2021 definition NUTS3 regional to make all the analyses. There were a total of 1514 NUTS3 regions of 37 European countries including UK (table A1). Our estimates were aggregated from NUTS3 to country and at four European regions level for each scenario and each general circulation model (GCM). European region wise classification of countries, their corresponding total NUTS3 regions are presented in table A1.

All the climate-related features were extracted from the E-OBS (1950-2014) and the ISIMIP-3b (2015-99) data of five primary general circulation models (GCMs) representing three probable futures with respect to climate and socio-economic changes.^5-8^ In correlative niche modelling the *bioclimatic* features are a commonly used set of time-varying geo-climatic metrics.^9^ Out of 19 bioclimatic features, 11 are derived from standard temperature variables, whereas the remaining are obtained from the precipitation for the region over time. As stated above, we first extracted and accumulated the grided temperature, precipitation, and relative humidity data spatially (NUTS3) and temporally (1950-99) for the study period. The data for population density and gross domestic product (GDP) at NUTS3 level was available and used from ISIMIP2b phase of CMIP6.

The features identifying the time and region-id were excluded from the analyses in order to avoid spatio-temporal autocorrelation that can artificially inflate a model performance. Moreover, it was done in order to make predictions purely based on the change of climatic, urbanization and socioeconomic conditions rather than inferring them from any encoded spatio-temporal information or patterns.^10^

Although the majority of the data for each predictor was complete, the missing observations (<1%) were imputed using the nearest-in-time available values of the same NUTS3 region for any predictor. The rest of the missing records were imputed using the country-level nearest-in-time values for each predictor.

Table A1- Summary of countries and NUTS3 regions per European region

| EU region | Total NUTS3/  region | Countries (ISO code) | Total NUTS3/ Country |
| --- | --- | --- | --- |
| Central and Eastern Europe | 259 | "AL”, "CZ", "EE”,"SI”,"BG" "HR","LV”,"MK”,"LT,"RO" "SK" (Total=11) | AL= 12, HU=180, PL=73, CZ= 30, EE=5, SI= 52, BG= 116, HR= 133, LV=6, MK= 8, LT= 10, RO=338, SK=16 |
| Northern Europe | 66 | "SE","DK", "FI", "IS”,"NO"  (Total=6) | SE= 21, DK=11, FI=19, IS=2, NO=13 |
| Southern Europe | 353 | "IT","TR", "ES","CY", "MT", "PT", “RS”, “EL”, “RS”,” ME”  (Total=10) | IT=467, TR=177, ES=83, CY=9, MT= 2, PT=33, RS=129, ME=1,  EL=53 |
| Western Europe | 836 | "DE", "IE", "FR", "CH", "AT", "BE", "NL", "LU", "LI",  “UK (Total=10) | DE= 505, IE=8, FR=165, CH= 26, AT=83, BE=44, NL=56, LU=1, LI=1, UK=179 |
| Europe | 815 | - | - |

# Model cross-validation and hyperparameter tuning

Machine learning models can be trained with different training and validation strategies like hold-out, *k-fold* cross-validation, and nested cross-validation. We opted for a more computationally expensive yet robust *k-fold* cross-validation approach to avoid any overfitting/underfitting. This was done for the training data set using 10-fold cross-validation. The model partitions the training data into *k-folds* (subsets) of equal size.^11^

The tree-based XGBoost comes with various hyperparameters, and the model performance can be improved by tuning and optimizing these parameters. While few parameters are general and depend on the nature of machine learning, others control the performance of the booster algorithm.^12^ Some of these hyperparameters are of importance while cross-validation is *nrounds*, which represents the number of trees to grow. One of the parameters, *eta,* controls the model’s learning rate from the data patterns. In contrast, *gamma* controls the regularization part of the cost function and is critical in preventing the model overfitting. The hyperparameter *min_child_weight is* important to block any potential feature interactions causing overfitting. Another crucial parameter is the *scale_pos_weight-* the ratio of positive classes to the negative classes to hand the class imbalance. Similarly, *lambda* is tuned during cross-validation and controls the *L2* regularization on weights. The hyperparameter *max_depth* controls the depth of the tree and is important to tune. Lastly, *max_delta_step* can also help in binary classification problems with extreme class imbalance*.*^12^

# Model classification metrics

**Threshold-independent metrics**

**Logloss:** *Logloss-score* is measure of how close a model predicted probability is to the corresponding actual/true class label (0 or 1 in case of binary classification). The more the predicted probability diverges from the actual value, the higher is the log-loss value. In essence, it means that if a model predicts an observation with label ‘0’ with very low probability (close to zero) and the observations with label ‘1’ with as higher probability as possible, the logloss score will decrease implying that the model

Mathematically, for a dataset has *N* number of observations and the class-label of observation ‘*i*’ is denoted by $y_{i}$( where $y_{i}$*=1* or $y_{i}$ *=0*) and its corresponding model predicted probability is *p_i_*, then the equation for *logloss-score* ($\mathbb{L}$) for the whole data set is given by

| $\mathbb{L=-}\frac{1}{N}\sum_{i=1}^{N} \left[ y_{i}lnp_{i}+\left( 1-y_{i} \right)\ln(1-p_{i}) \right],$ | S1 |
| --- | --- |

AUC score: The AUC (area under the Receiver operator characteristic/precision recall curve) score is indicative of degree of separability/distinction or intermingling/crossover between the predictions of the two classes (here, regions with (1) and without (0) WNV transmission). It is the probability that the model ranks a randomly chosen WNV positive region higher than a randomly chosen WNV negative region. The area under the receiver operator characteristic (AUROC) curve (or precision-recall characteristic (AUPRC) curve) is another very commonly used metric to quantify a classification-based model’s performance. Compared to AUPRC, the AUROC curve is more commonly used metric. Higher the score, higher the distinction and lower the crossover of the predictions of the two classes. In our setting for projections, we these scores were estimated from the ROC/PRC curve generated using the *confidence-based performance estimation* (CBPE) method.^13^

**Threshold-dependent metrics**

Threshold-dependent metrics of a classification model are those computed based on the confusion-matrix. Most commonly used of these metrics include *accuracy*, *sensitivity* (or *true-positive rate (TPR)*), *specificity* (*1-False-positive rate (FPR)*), and *precision* (*positive-prediction rate (PPR)*), *F1-score* and so on.

Here, we briefly describe some of the metrics important related to this study.

**Sensitivity/Recall:** *Sensitivity* or recall metric is the ratio of correctly predicted WNV positive (TP) regions to all regions that are actually WNV positive*.* This estimate measures how many of the actual positive instances we were able to correctly predict (or *recall*). This metric becomes important when we believe False Negatives are more important than False Positives.

| $Sensitivity=\frac{TP}{TP+FN},$ | S2 |
| --- | --- |

**Specificity:** Specificity metric is the ratio of correctly predicted WNV negative regions (TN) to all regions that are actually WNV negative*.* It measures how many of the actual negative instances we were able to correctly predict.

| $Specificity=\frac{TN}{TN+FP},$ | S3 |
| --- | --- |

**Precision:** Precision is the ratio of correctly predicted WNV positive regions to all the predicted to be positive of a confusion matrix. Intuitively, precision, as the name suggests, represents how precise, are our model's predictions of WNV positive regions.

| $Precision=\frac{TP}{TP+FP}$ | S5 |
| --- | --- |

**F1-Score:** F1-score/ F-Measure is a single performance metric that takes both sensitivity and precision into account. It is estimated by taking the harmonic mean of the two metrics:

| $F1=\frac{2.Precision.Recall}{Precision+Recall}$ | S6 |
| --- | --- |

F1-score will be higher only when both the precision and recall metrics have good performance.

In this study, due to high class-imbalance the in favor of regions with no reported WNV infections (WNV negative regions) (96:04) during the internal validation period, it was not appropriate to judge the model performance with accuracy as it can falsely depict a positive picture of the model predictions when in fact it does no good job than a random classifier. Hence, we examined the model’s predictive power with various other metrics described above that include both threshold-independent as well as threshold-dependent types. For threshold-dependent metrics, we evaluated the metrics finding an optimal classification-threshold since the general threshold of 0.5 can depict a negative predictive picture of a model when, indeed, it does a decent to very good job.

**Confidence-Based Performance Estimation (CBPE) method**

The CBPE framework is based on the performance of a classifier to accurately assign the correct class labels to out-of-sample data set assuming that its expected performance is extracted from the calibrated probabilities of the predicted scores.

Thus, to apply the method, the scores/probabilities of classifier must be calibrated first. Indeed, the outputs from the XGBoost-like classification algorithms cannot be treated as the true class probabilities since during the fitting procedure they prioritize the minimization of loss function putting less importance on calibrating them correctly. Consequently, the model outputs are required to be calibrated before applying the CBPE framework through an intermediate calibration classifier. Two most commonly used calibration classifiers include the *logistic regressor* (also known as Platt scaling) and the *isotonic regressor*. Here, we used the logistic regressor as calibration classifier. Next, it is assumed that there is no concept-drift between the training and the out-of-sample data. If present, the performance metrics may deteriorate largely because the method estimates the performance owing to the population shift or data drift. On the other hand, the method can be used to evaluate a classifier’s performance in absence of ground-truth when there exists a data drift, i.e., change in the distribution of the predictors between training and test data.

The CBPE method uses the calibrated probabilities of a classifier as input to assign a correct class to the out-of-sample data with the expectation that the quality of the model can be determined by the class probabilities. Similar to the hard coded labels the expected probabilities are summed by choosing a classification threshold. Assuming that the classification threshold is choose as $t$ and the model calibrated probabilities are denoted by $\hat{p}$, the expected metrics of the confusion matrix of the data without ground-truth can be estimated using the equations. (S7-S10) as:

|  |  |
| --- | --- |
| \| $\tilde{TP}=\sum\hat{p}\vert\hat{p}\geq t$ \| S7 \| \| --- \| --- \| |  |
| \| $\tilde{TN}= \sum1-\hat{p}\vert\hat{p}<t$ \| S8 \| \| --- \| --- \|  \| $\tilde{FP}=\sum1-\hat{p} \vert\hat{p}\geq t$ \| S9 \| \| --- \| --- \|  \| $\tilde{FN}=\sum\hat{p}\vert\hat{p}<t$ \| S10 \| \| --- \| --- \| |  |

Where $\tilde{TP}, \tilde{TN}$, $\tilde{FP}$ and $\tilde{FN}$ represent the expected true positive, true negative, false positive and false negative scores, respectively.

We used the above sets of equations to estimate all the performance metrics of our model for future data (see next section and table-A2)

While it can be crucial to examine all the assumptions of CBPE method, however, it requires independent but more rigorous analysis as was performed recently in literature in the settings for an astronomical data set.^14^ Since, the aim of this analysis was not to thoroughly examine the robustness of the CBPE method in our setting but to ensure that the overall results remain stable, we omit those analysis for a future work. The fact that overall model predicted trends (figure A3) from the current period quite find the positive correlative relationship between climatic conditions and the WNV outbreaks validates the fact the model assumptions are satisfied well.

# Performance metrics on out-of-sample data

The overall predictive power of the algorithm during internal period remained consistently very high (figure 1, Train). Similarly, the model performance estimated using the CBPE method showed promising results (figure1, Test). An average AUROC-score of 0.84 (range: 0.78-0.90) was achieved for the test data. The optimal classification-threshold from ROC-curve produced a sensitivity score of 0.7 (range: 0.6-0.84), and specificity score of 0.92 (range: 0.90-0.94). Similarly, the average AUPRC-score was 0.50 (range: 0.69-0.76) (figure A1; B). This score apparently may not look convincing shows that the classifier did a decent job despite the sever class-imbalance (96:04) in favor of negative instances. The extent of class-imbalance a classifier predicting any score above 0.04 is doing making some contribution. The fact that the baseline score (i.e., the proportion of positive instances in the training data) was only 4%, shows that the performance it remains consistent with that of to a random classifier. A summary of the performance metrics of each all models is presented in table A2.

**Figure A1: Receiver-operator characteristic (ROC) curves**

**A)** ROC-curves of each climate model estimated of training (2010-22) and test periods (1950-2009 and 2023-99). The dashed diagonal line shows the random classifier. The expected ROC curve for the test period was estimated using the confidence-based performance estimation (CBPE) methodology.

**Table A2**: External validation performance metrics for optimal threshold of the models

| **Threshold** | **TPR** | **FPR** | **PPR** | **F1** | **AUC**  **score** | **Metric**  **type** | **SSP-RCP** | **Climate**  **Model** |
| --- | --- | --- | --- | --- | --- | --- | --- | --- |
| 0.04 | 0.63 | 0.06 | 0.29 | 0.40 | 0.81 | ROC | SSP-RCP126 | GFDL-ESM4 |
| 0.26 | 0.50 | 0.02 | 0.56 | 0.53 | 0.46 | PRC | SSP-RCP126 | GFDL-ESM4 |
| 0.05 | 0.73 | 0.07 | 0.35 | 0.47 | 0.86 | ROC | SSP-RCP370 | GFDL-ESM4 |
| 0.29 | 0.59 | 0.02 | 0.59 | 0.59 | 0.54 | PRC | SSP-RCP370 | GFDL-ESM4 |
| 0.05 | 0.72 | 0.08 | 0.33 | 0.45 | 0.85 | ROC | SSP-RCP585 | GFDL-ESM4 |
| 0.29 | 0.57 | 0.02 | 0.58 | 0.58 | 0.53 | PRC | SSP-RCP585 | GFDL-ESM4 |
| 0.04 | 0.65 | 0.07 | 0.27 | 0.38 | 0.82 | ROC | SSP-RCP126 | IPSL-CM6A-LR |
| 0.26 | 0.50 | 0.02 | 0.53 | 0.51 | 0.45 | PRC | SSP-RCP126 | IPSL-CM6A-LR |
| 0.05 | 0.68 | 0.08 | 0.29 | 0.41 | 0.83 | ROC | SSP-RCP370 | IPSL-CM6A-LR |
| 0.26 | 0.52 | 0.02 | 0.52 | 0.52 | 0.47 | PRC | SSP-RCP370 | IPSL-CM6A-LR |
| 0.06 | 0.75 | 0.09 | 0.34 | 0.46 | 0.87 | ROC | SSP-RCP585 | IPSL-CM6A-LR |
| 0.29 | 0.59 | 0.03 | 0.56 | 0.57 | 0.53 | PRC | SSP-RCP585 | IPSL-CM6A-LR |
| 0.03 | 0.58 | 0.07 | 0.23 | 0.33 | 0.78 | ROC | SSP-RCP126 | MPI-ESM1-2-HR |
| 0.24 | 0.44 | 0.01 | 0.54 | 0.49 | 0.41 | PRC | SSP-RCP126 | MPI-ESM1-2-HR |
| 0.04 | 0.66 | 0.08 | 0.26 | 0.37 | 0.82 | ROC | SSP-RCP370 | MPI-ESM1-2-HR |
| 0.25 | 0.50 | 0.02 | 0.52 | 0.51 | 0.45 | PRC | SSP-RCP370 | MPI-ESM1-2-HR |
| 0.04 | 0.70 | 0.09 | 0.27 | 0.39 | 0.84 | ROC | SSP-RCP585 | MPI-ESM1-2-HR |
| 0.27 | 0.53 | 0.02 | 0.55 | 0.54 | 0.49 | PRC | SSP-RCP585 | MPI-ESM1-2-HR |
| 0.03 | 0.60 | 0.06 | 0.26 | 0.36 | 0.79 | ROC | SSP-RCP126 | MRI-ESM2-0 |
| 0.26 | 0.47 | 0.01 | 0.58 | 0.52 | 0.45 | PRC | SSP-RCP126 | MRI-ESM2-0 |
| 0.04 | 0.66 | 0.07 | 0.29 | 0.40 | 0.82 | ROC | SSP-RCP370 | MRI-ESM2-0 |
| 0.27 | 0.52 | 0.02 | 0.56 | 0.54 | 0.47 | PRC | SSP-RCP370 | MRI-ESM2-0 |
| 0.04 | 0.67 | 0.08 | 0.27 | 0.38 | 0.83 | ROC | SSP-RCP585 | MRI-ESM2-0 |
| 0.26 | 0.51 | 0.02 | 0.53 | 0.52 | 0.46 | PRC | SSP-RCP585 | MRI-ESM2-0 |
| 0.05 | 0.72 | 0.08 | 0.32 | 0.44 | 0.85 | ROC | SSP-RCP126 | UKESM1-0-LL |
| 0.28 | 0.56 | 0.02 | 0.56 | 0.56 | 0.51 | PRC | SSP-RCP126 | UKESM1-0-LL |
| 0.08 | 0.83 | 0.10 | 0.42 | 0.55 | 0.90 | ROC | SSP-RCP370 | UKESM1-0-LL |
| 0.32 | 0.68 | 0.04 | 0.61 | 0.64 | 0.63 | PRC | SSP-RCP370 | UKESM1-0-LL |
| 0.06 | 0.74 | 0.10 | 0.31 | 0.44 | 0.86 | ROC | SSP-RCP585 | UKESM1-0-LL |
| 0.04 | 0.27 | 0.56 | 0.03 | 0.54 | 0.55 | PRC | SSP-RCP585 | UKESM1-0-LL |
|  |  |  |  |  |  |  |  |  |

**Note:** *AUC score represents both AUROC and AUPRC scores*

# Figure A2: Simulated changes in the WNV expansion risk: The model predicted WNV risk probability maps as ensemble mean across the climate models at four time periods; the past (1950-70), the current (2000-20) and future projections (2040-60 and 2080-00) under each climate scenario.

# Europe and country-wise risk trends

In addition to the European-regions analyses presented in the main text, here we show a general picture of the virus risk overall for whole Europe. Specifically, the trends of highly correlated maximum spring temperature (^o^C) and relative humidity (%) predictors and the model-estimated risk are shown from 1950 to 2100 in figure A3.

The new areas were classified based on the optimal PRC-threshold only in order to reduce the false discovery rate (FDR). In figure A3, country-wise WNV outbreak temporal risk trends are shown from 1950 to 2100 for each climatic scenario.

**Heterogeneous country-wise WNV risk projections**

Country-level analyses exhibited heterogeneous disease risk forecasts in the coming decades (Appendix; figure A4). Evidently, most Western European countries would undergo a tangible rise in WNV risk under the considered scenarios if no interventions were implemented. The virus expansion to these countries is much likely to be a reality since it already circulates in parts of Germany,^15,16^ the Netherlands,^17^ and its competent vector, *Culex modestus,* are found in Belgium.^18^ On the other hand, currently endemic South-eastern and Central European countries might have reached a climatic threshold under some scenarios later in this century where the risk might decrease due to the conditions being too hot for vectors to survive. However, countries like Poland, Czech Republic, Portugal, and Slovakia etc., are all expected to experience a substantial increase in terms of potential outbreak-prone areas. The risk for Northern European increased many-fold but only under medium to high mitigation and CO_2_ emissions scenarios underpinning the adverse climate impacts.


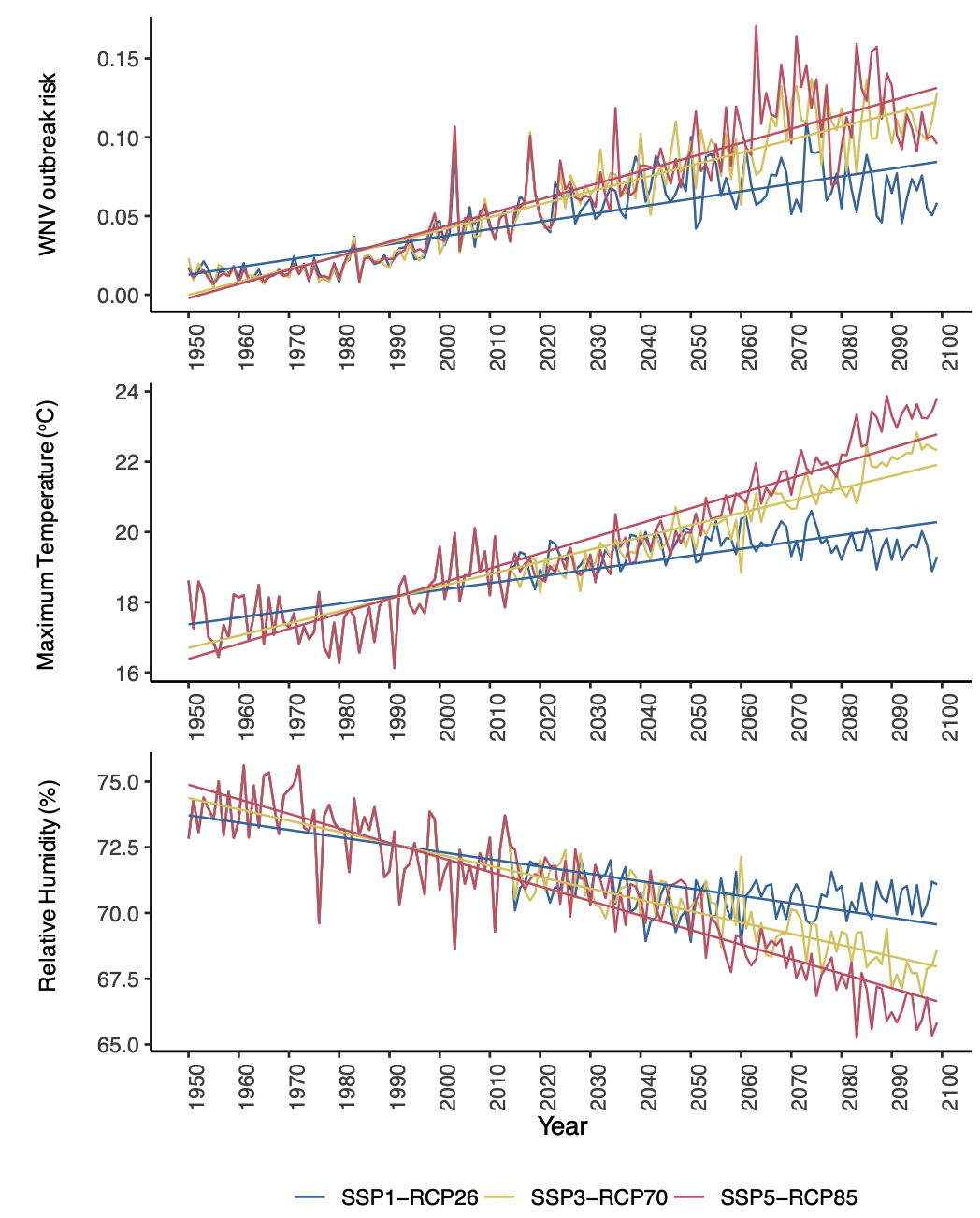


**Figure A3:** Annual trends in model predicted WNV outbreak risk, the maximum spring temperature and relative humidity of spring for Europe across each scenario.

**Figure A4:** Country-wise WNV-outbreak risk trends for under each climate scenario. The estimate shown are smooth representation inferred from the regression fit of the ensemble means across all the circulation models.

**Statistical correlations analysis**

Pearson correlations of predictors with model predicted outbreak risk were performed under each SSP-RCP in order to identify important climatic variables in context of disease early warning alert. For the temperature related features, we chose maximum spring temperature (*max_temp_Q02*) although summer temperatures (*bio5*, *bio10*) were equivalently highly correlated. Higher than normal spring temperatures have been shown to lengthen transmission season, and they provide early signal before the actual transmission starts as found previously.^19^ Hence, we emphasized that the spring climate is an important climatic alert for an outbreak.

**Figure A5:** Correlation coefficients of the model predicted WNV outbreak risk associated with each of the covariates across all scenarios.

# Reference

1. European Center for Disease Prevention and Control. <https://www.ecdc.europa.eu/en>. (accessed November 16 2021).

2. Liu Y, Li Y, Li S, Motesharrei S. Spatial and temporal patterns of global NDVI trends: correlations with climate and human factors. *Remote Sensing* 2015; **7**(10): 13233-50.

3. Lahti L, Huovari J, Kainu M, Biecek P. Retrieval and Analysis of Eurostat Open Data with the eurostat Package. *R J* 2017; **9**(1): 385.

4. Watts MJ, Sarto IMV, Mortyn PG, Kotsila P. The rise of West Nile Virus in Southern and Southeastern Europe: A spatial-temporal analysis investigating the combined effects of climate, land use and economic changes. *One Health* 2021; **13**: 100315.

5. Lange S. Trend-preserving bias adjustment and statistical downscaling with ISIMIP3BASD (v1.0). *Geosci Model Dev* 2019; **12**(7): 3055-70.

6. Eyring V, Bony S, Meehl GA, et al. Overview of the Coupled Model Intercomparison Project Phase 6 (CMIP6) experimental design and organization. *Geosci Model Dev* 2016; **9**(5): 1937-58.

7. Meinshausen M, Nicholls ZRJ, Lewis J, et al. The shared socio-economic pathway (SSP) greenhouse gas concentrations and their extensions to 2500. *Geosci Model Dev* 2020; **13**(8): 3571-605.

8. Cornes RC, van der Schrier G, van den Besselaar EJ, Jones PD. An ensemble version of the E‐OBS temperature and precipitation data sets. *Journal of Geophysical Research: Atmospheres* 2018; **123**(17): 9391-409.

9. Kriticos DJ, Jarošik V, Ota N. Extending the suite of bioclim variables: a proposed registry system and case study using principal components analysis. *Methods in Ecology and Evolution* 2014; **5**(9): 956-60.

10. Hijmans R, Phillips S, Elith J. J (2017) dismo: Species Distribution Modeling. R package version 1.1-4.

11. Farooq Z, Rocklov J, Wallin J, et al. Artificial intelligence to predict West Nile virus outbreaks with eco-climatic drivers. *Lancet Reg Health Eur* 2022; **17**: 100370.

12. Chen T, Guestrin C. Xgboost: A scalable tree boosting system. Proceedings of the 22nd acm sigkdd international conference on knowledge discovery and data mining; 2016; 2016. p. 785-94.

13. NannyML. Confidence-based Performance Estimation (CBPE). 2022. <https://nannyml.readthedocs.io/en/stable/how_it_works/performance_estimation.html> (accessed 05 August 2022).

14. Humphrey A, Kuberski W, Bialek J, et al. Machine-learning classification of astronomical sources: estimating F1-score in the absence of ground truth. *Monthly Notices of the Royal Astronomical Society: Letters* 2022; **517**(1): L116-L20.

15. Ziegler U, Santos PD, Groschup MH, et al. West Nile virus epidemic in Germany triggered by epizootic emergence, 2019. *Viruses* 2020; **12**(4): 448.

16. Pietsch C, Michalski D, Munch J, et al. Autochthonous West Nile virus infection outbreak in humans, Leipzig, Germany, August to September 2020. *Euro Surveill* 2020; **25**(46).

17. Vlaskamp DR, Thijsen SF, Reimerink J, et al. First autochthonous human West Nile virus infections in the Netherlands, July to August 2020. *Eurosurveillance* 2020; **25**(46): 2001904.

18. Wang L, Rosales Rosas AL, De Coninck L, et al. Establishment of Culex modestus in Belgium and a glance into the virome of Belgian mosquito species. *Msphere* 2021; **6**(2): e01229-20.

19. Marini G, Manica M, Delucchi L, Pugliese A, Rosa R. Spring temperature shapes West Nile virus transmission in Europe. *Acta Trop* 2021; **215**: 105796.
